# *APOL1* risk alleles among individuals with CKD in Northern Tanzania: A pilot study

**DOI:** 10.1371/journal.pone.0181811

**Published:** 2017-07-21

**Authors:** John W. Stanifer, Francis Karia, Venance Maro, Kajiru Kilonzo, Xuejun Qin, Uptal D. Patel, Elizabeth R. Hauser

**Affiliations:** 1 Department of Medicine, Duke University; Durham, NC United States of America; 2 Duke Global Health Institute, Duke University; Durham, NC United States of America; 3 Duke Clinical Research Institute, Duke University; Durham, NC United States of America; 4 Kilimanjaro Christian Medical College; Moshi, Tanzania; 5 Duke Molecular Physiology Institute, Duke University School of Medicine; Durham NC United States of America; 6 Department of Biostatistics and Bioinformatics, Duke University School of Medicine; Durham, NC United States of America; Case Western Reserve University, UNITED STATES

## Abstract

**Introduction:**

In sub-Saharan Africa, approximately 100 million people have CKD, yet genetic risk factors are not well-understood. Despite the potential importance of understanding *APOL1* risk allele status among individuals with CKD, little genetic research has been conducted. Therefore, we conducted a pilot study evaluating the feasibility of and willingness to participate in genetic research on kidney disease, and we estimated *APOL1* risk allele frequencies among individuals with CKD.

**Methods:**

In 2014, we conducted a community-based field study evaluating CKD epidemiology in northern Tanzania. We assessed for CKD using urine albumin and serum creatinine to estimate GFR. We invited participants with CKD to enroll in an additional genetic study. We obtained dried-blood spots on filter cards, from which we extracted DNA using sterile punch biopsies. We genotyped for two single nucleotide polymorphisms (SNPs) defining the *APOL1* G1 risk allele and an insertion/deletion polymorphism defining the G2 risk allele. Genotyping was performed in duplicate.

**Results:**

We enrolled 481 participant, 57 (12%) of whom had CKD. Among these, enrollment for genotyping was high (n = 48; 84%). We extracted a median of 19.4 ng of DNA from each dried-blood spot sample, and we genotyped the two *APOL1* G1 SNPs and the *APOL1* G2 polymorphism. Genotyping quality was high, with all duplicated samples showing perfect concordance. The frequency of *APOL1* risk variants ranged from 7.0% to 11.0%, which was similar to previously-reported frequencies from the general population of northern Tanzania (p>0.2).

**Discussion:**

In individuals with CKD from northern Tanzania, we demonstrated feasibility of genotyping *APOL1* risk alleles. We successfully genotyped three risk variants from DNA extracted from filter cards, and we demonstrated a high enrollment for participation. In this population, more extensive genetic studies of kidney disease may be well-received and will be feasible.

## Introduction

Chronic kidney disease (CKD) is a growing global burden that disproportionately affects people in low- and middle-income countries [1, 2]. In sub-Saharan Africa, the prevalence may be as high as 14%, with as many as 100 million people living with CKD [3]. Nonetheless, risk factors for CKD remain poorly characterized in the vast, heterogeneous region comprising myriad tribal and ethnic populations [2, 4]. In this context, the importance of genetic risk factors for CKD is gaining attention as possible key contributors to the growing burden of CKD in the region [5].

Among the potentially most important genetic risk factors for CKD in sub-Saharan Africa are the *APOL1* risk alleles [6]. *APOL1* is protective against several African trypanosomes which cause Africa sleeping sickness [7–9]. This protective effect has been speculated as a cause for positive selection in African populations, with the *APOL1*G1 and G2 variants occurring in highest frequencies in western African populations [6, 10, 11]. The risk alleles have also been reported with variable frequency in central African populations among many of Bantu ancestry, where African sleeping sickness remains highly endemic [12–14].

On the other hand, few studies have investigated *APOL1* carrier status among east African populations who have shared ancestries but different infectious and environmental exposures. For example, the known observed frequency of *APOL1* is highest in the western and central continent where *Trypanosoma brucei gambiense* mostly occurs. However, *APOL1* appears to be most trypanolytic against *Trypanasoma brucei rhodesiense*, which occurs primarily in the eastern continent [6, 10, 13], and although the prevalence of *APOL1* risk alleles appears to be lower in many east African populations, the relationship between these alleles and CKD has not been widely explored [6, 12]. The Human Hereditary and Health in African Kidney Disease Research Network (H3AFRICA) Study is broadly seeking to explore this relationship in many populations, including east African populations, but recruitment has been challenging in some populations, particularly among those with more advanced kidney disease [5, 15]. Therefore, as part of the Comprehensive Kidney Disease Assessment for Risk Factors, epidemiology, Knowledge, and Attitudes (CKD-AFRiKA) Study [16–18], we conducted a pilot study to: 1) evaluate willingness to participate in genetic research studies among individuals with CKD in an East African setting; 2) demonstrate feasibility of genotyping *APOL1* risk alleles G1 and G2 from dried-blood spot samples on filter cards obtained during this field; and 3) assess the frequency of *APOL1* risk variants among individuals with CKD from northern Tanzania.

## Methods

### Ethics statement

The study protocol was approved by Duke University Institutional Review Board (#Pro00040784), the Kilimanjaro Christian Medical College (KCMC) Ethics Committee (EC#502), and the National Institute for Medical Research (NIMR) in Tanzania. Written informed consent (by signature or thumbprint) was obtained from all participants, and all participants with abnormal findings received counseling, educational pamphlets, and reimbursement with referral for follow-up.

### Study setting

We conducted a stratified, cluster-designed cross-sectional field study between January and June 2014 in the Kilimanjaro Region of northern Tanzania. The adult regional population is more than 900,000 people. It has a female majority (58%), and 65% of the adult population lives in a rural setting. The largest ethnic group is the Chagga tribe (60%), who are primarily of Bantu ancestry and the third largest ethnic group in Tanzania. Other major tribes in the region include the Pare, Sambaa, and Maasai. The region comprises seven districts; our study was conducted in the Moshi Urban and Moshi Rural districts [19]. We have previously reported the prevalence of CKD to be 7.0% (95% CI 3.8–12.3)[17].

### Sampling

Detailed sampling methods for the CKD-AFRiKA Study have been previously reported [17, 20]. In brief, we used a three-stage cluster probability sampling method, stratified by urban and rural setting, to randomly select households from randomly selected neighborhoods in the Moshi Urban and Moshi Rural districts. All non-pregnant, community-dwelling adults (age ≥ 18 years old) from the representative households were recruited into the study. The sample size was designed to estimate the prevalence of CKD with a precision of 5% when accounting for the cluster-design effect. To reduce non-response rates, we attempted a minimum of two additional visits during off-hours (evenings and weekends) and multiple phone calls using mobile phone numbers. For all enrolled participants, we collected demographic data, self-reported medical history, and anthropomorphic data.

### CKD assessment

As part of the CKD AFRiKA study, all enrolled participants were assessed for CKD using serum creatinine and urine albumin [17]. The serum creatinine was measured using the Cobas Integra 400 Plus (Roche Diagnostics; Basel Switzerland) at the Kilimanjaro Christian Research Institute Biotechnology Laboratory. The laboratory is known in the region for its high quality results, and it participates fully in international external quality assurance programs including the College of American Pathologists and the United Kingdom External Quality Assessment Service. All laboratory investigations were conducted according to Good Clinical Laboratory Practice standards. Urine albumin was detected with a Siemens MicroAlbustix (Siemens Healthcare Diagnostics, Inc.; Tarrytown, NY) from a mid-stream urine sample. All positive urine albumin results were confirmed with a repeat measurement from a first-morning void, mid-stream urine sample more than 48 hours from the initial measurement.

CKD was defined according to the Kidney Disease Improving Global Outcomes Working Group guidelines [21]. To estimate glomerular filtration rate (eGFR), we used the Modification of Diet in Renal Disease (MDRD) equation without the race factor. Stage I was defined as albuminuria with an eGFR greater than 90 ml/min/1.73m^2^, Stage II as albuminuria with an eGFR between 60 and 90 ml/min/1.73m^2^, Stage III as an eGFR between 30 and 60 ml/min/1.73m^2^ with or without albuminuria, Stage IV as an eGFR between 15 and 30 ml/min/1.73m^2^ with or without albuminuria, and Stage V as an eGFR less than 15 ml/min/1.73m^2^ with or without albuminuria. A urine albumin greater than 30 mg/dL in the absence of gross hematuria or an ongoing urinary tract infection as confirmed by urinalysis (Siemens Multistix 10G Urinalysis test strips) was considered positive. Quality control measures were performed weekly on each open and new bottle of urine dipsticks according to the manufacturer’s recommendation.

### Dried blood spot collection and handling

We obtained dried-blood spots to assess *APOL1* status for participants with CKD. Dried-blood spot samples, obtained by fingerprick, were spotted (up to 125μL per each 1 inch application) on Whatman FTA® cards (Whatman Inc., Brentford UK) using capillary tubes and subsequently dried at room temperature for 1 hour. Whatman FTA® cards are designed for room temperature collection, shipment, archiving, and purification of nucleic acids from human specimens. We stored and transported the spotted Whatman FTA® cards at room temperature (20–25° C) with a desiccant for humidity-control.

### DNA extraction

Using sterile biopsy punches, we took 6mm punches of each dried-blood spot sample. The filter card punch was placed directly into an extraction tube, and the DNA was isolated on the Qiagen Autopure (Qiagen Inc., Germantown, MD), using PureGene chemistry. We quantified DNA using PicoGreen dsDNA Assays (Thermo Fisher Scientific, Waltham, MA). Given the small elution volumes (20 μL), we stored the DNA at -80° C prior to genotyping. We assessed quality using the Agilent TapeStation (Agilent Technologies Inc., Santa Clara CA), and each DNA sample was assigned an Extraction Quality (EQ) score (range: 1–5) to indicate the average fragment length.

### Genotyping

Two single nucleotide polymorphisms (SNPs) in the *APOL1* G1 nephropathy risk locus (rs73885319; rs60910145) were genotyped on the Applied Biosytems 9700 384 Well Dual Block GeneAmp Thermocyclers, and an insertion/deletion polymorphism for the G2 risk locus (rs71785313) was genotyped on the Applied Biosystems ViiA-7 Real Time PCR System. Scatter plots of genotypes calls were examined to evaluate clustering. Additional quality control measures included genotyping of two negative controls (blank wells) for each SNP and two Centre d’Etude du Polymorphism Humain (CEPH) controls. Every study sample was duplicated for each SNP to evaluate genotyping quality.

### Statistical analyses

Data were analyzed using STATAv.14 (STATA Corp., College Station, TX) and R statistical software packages [22]. The mean and standard deviation (SD) or median and inter-quartile ranges (IQR) were reported for continuous variables. Categorical variables were summarized using counts and percentages. We obtained allele and genotype frequencies and tested Hardy-Weinberg Equilibrium by comparing expected and observed genotype frequencies. We used the two sample *z*-test approximation to the binomial to compare allele frequencies between the CKD-AFRiKA Study and African populations. To ensure a dataset of genetically independent individuals, we excluded an offspring from a parent-offspring pair in data analyses. All p values are two-sided at a 0.05 significance level. We obtained population *APOL1* risk allele frequencies from the Allele Frequency Database [23], which included *APOL1* risk allele frequencies for ethnically Chagga populations with unknown CKD status from the Kilimanjaro Region in northern Tanzania and other Bantu and non-Bantu populations from central and eastern Africa with unknown CKD status.

## Results

Between January and June 2014, we enrolled 481 adults into the CKD-AFRiKA Study, of whom 57 (12%) were identified as having CKD (**Table 1**). Of the 57 participants with CKD, enrollment into the genotyping studies was high, with most participants (n = 48; 84%) agreeing to participate.

**Table 1:** Characteristics of participants with CKD, by genotyping status; n = 57.

Variable	Overall (n = 57)	Agreed to Genotyping (n = 48)	Not Genotyped (n = 9)	p-value*
**Gender**				0.180
**Male**	17 (30%)	16 (33%)	1 (11%)	
**Female**	40 (70%)	32 (67%)	8 (89%)	
**Age**				0.491
**18–39 years old**	16 (28%)	12 (25%)	4 (44%)	
**40–59 years old**	24 (42%)	21 (44%)	3 (33%)	
**60+ years old**	17 (30%)	15 (31%)	2 (22%)	
**Setting**				0.392
**Rural**	3 (5%)	2 (4%)	1 (11%)	
**Urban**	54 (95%)	46 (96%)	8 (89%)	
**Ethnicity**				0.422
**Chagga**	37 (65%)	31 (65%)	6 (67%)	
**Pare**	7 (12%)	5 (10%)	2 (22%)	
**Sambaa**	4 (7%)	3 (6%)	1 (11%)	
**Other‡**	9 (16%)	9 (19%)	0 (0%)	
**Education**				0.145
**None**	4 (7%)	2 (5%)	2 (22%)	
**Primary**	40 (70%)	36 (75%)	4 (44%)	
**≥Secondary**	13 (23%)	10 (20%)	3 (33%)	
**Occupation**				0.292
**Unemployed**	10 (17%)	10 (21%)	0 (0%)	
**Farmer/Wage Earner**	14 (25%)	11 (23%)	3 (33%)	
**Small Business/Vendors**	21 (37%)	16 (33%)	5 (56%)	
**Professional**	12 (21%)	11 (23%)	1 (11%)	
**CKD Stage**				0.535
**Stage I/II**	43 (75%)	37 (77%)	6 (66%)	
**Stage III**	12 (21%)	9 (19%)	3 (33%)	
**Stage IV/V**	2 (4%)	2 (4%)	0 (0%)	

‡Other ethnicities includes Maasai, Luguru, Kilindi, Kurya, Mziguwa, Mnyisanzu, Rangi, Jita, Nyambo, and Kaguru

*p-value comparing participants genotyped versus those not genotyped

Among the 48 participants who agreed to genotyping, most were urban residents (n = 46; 96%), female (n = 32; 67%), ethnically Chagga (n = 31; 65%), had a primary school education (n = 36; 75%) and worked in a self-employed small business/vendor (n = 16; 33%). The median age was 51.5 years (IQR: 40–65). Most participants with CKD were classified as Stage I or II (n = 37; 77%), with fewer participants having Stage III (n = 9; 19%), or Stage IV/V (n = 2; 4%). We observed no significant differences in the sociodemographics or CKD severity between the participants enrolled in the genotyping studies and those not enrolled (p>0.10 for all).

We extracted a median of 19.4 ng of DNA from the dried-blood spot samples. The range of DNA extracted was 4.4ng to 725ng, with all but four filter cards yielding >10.0 ng. The mean EQ score for DNA fragment length was 2.91 (SD 0.35). We successfully genotyped two SNPs (rs73885319; rs60910145) in the *APOL1* G1 allele, and we genotyped one indel (rs71785313) in the *APOL1* G2 allele. We had one participant with a missing genotype for rs60910145 due to failure of both duplicated samples. For the other two markers (rs71785313 and rs73885319) one duplicate for each failed to return a genotype. The genotyping plots demonstrated discrete clusters for the intensities of each allele (**S1 Fig**). Genotyping quality was high; for all samples with both duplicated genotypes there was perfect concordance. We observed strong linkage disequilibrium between the *APOL1* G1 defining alleles (r^2^ of 0.930), and the *APOL1* G2 allele demonstrated linkage equilibrium with both of the *APOL1* G1 SNPs, with r^2^ of 0.001 and 0.020 for rs73885319 and rs60910145, respectively. Two of the three genotyped *APOL1* SNPs were in Hardy-Weinberg equilibrium, and one (rs60910145) deviated slightly (p = 0.0078). The analysis of allele frequencies included 47 genetically independent participants due to removal of an offspring from a parent-offspring pair. The frequency of the *APOL1* G1 and *APOL1* G2 risk allele SNP variants ranged from 7.0% to 11.0% (**Table 2**). We observed no significant difference (p>0.2 for all) in the frequency of these risk variants from previously-reported frequencies from northern Tanzanian populations [23].

**Table 2:** Chromosome 22 APOL1 genotyping results.

Gene	SNP	C22 position (GRCh37)	Risk Allele	Risk allele frequency, cases	Risk allele frequency, reference population* (95% CI)	P-value
*APOL1*G1	rs60910145	36662034	G	0.11	0.11 (0.07, 0.17)	0.96
*APOL1*G1	rs73885319	36661906	G	0.09	0.14 (0.09, 0.20)	0.28
*APOL1*G2	rs71785313	36662051	C	0.07	0.08 (0.05, 0.13)	0.86

*Reference population included ethnically Chagga populations from the Kilimanjaro Region in northern Tanzania obtained through the Allele Frequency Database.

In our study population, which consisted predominantly of Chagga individuals of Bantu ancestry, the frequency of *APOL1* G1 risk variants was significantly higher than that reported for other Central African Bantu tribes, the Biaka and Mbuti tribes and a non-Bantu Tanzanian tribe, the Sandawe tribe of Khoisan ancestry (p<0.01 for all) (**Table 3**)[23–28]. On the other hand, we observed a significantly lower frequency for the *APOL1* G1 risk variants compared with a Kenyan Bantu tribe, the Luhya (p<0.01) and a slightly lower frequency compared with another Tanzanian Bantu tribe, the Zaramo. We observed a similar frequency to the Maasai, a non-Bantu Tanzanian tribe of Nilotic ancestry (p>0.5). For the *APOL1* G2 risk variant, we observed a frequency similar to the Biaka, Mbuti, Sandawe, and Maasai tribes (p>0.5 for all), and a significantly lower frequency than the Zaramo tribe (p = 0.02).

**Table 3:** Frequency of *APOL1* G1 and *APOL1* G2 risk variants for CKD-AFRiKA study sample and other populations of interest.

Ancestry	Setting	Tribe	Sample Size (N)	Risk Variant Frequency (95% CI)		
				*APOL1* G1		*APOL1* G2
				rs60910145	rs73885319	rs71785313
**Bantu**	CKD-AFRiKA (Tanzania)	Chagga	47	0.11 (0.07, 0.16)	0.09 (0.06, 0.15)	0.07 (0.04, 0.12)
	Central African	Biaka	138	**0.01 (0.01, 0.04)**	**0.01 (0.01, 0.04)**	0.08 (0.05, 0.12)
		Mbuti	78	**0.01 (0.00, 0.05)**	**0.01 (0.00, 0.05)**	0.03 (0.01, 0.07)
	Tanzania	Zaramo	66	0.17 (0.11, 0.24)	0.17 (0.11, 0.24)	**0.15 (0.10, 0.23)**
	Kenya	Luhya	226	**0.24 (0.21, 0.29)**	**0.24 (0.21, 0.29)**	N/A
**Nilotic**	Tanzania	Maasai	40	0.08 (0.03, 0.16)	0.10 (0.04, 0.19)	0.10 (0.05, 0.19)
**Khoisan**	Tanzania	Sandawe	80	**0 (0)**	**0 (0)**	0.09 (0.05, 0.15)

BOLD indicates significant frequency risk difference (p<0.05) compared with CKD-AFRiKA population

## Discussion

From a community-based study of predominantly Bantu-ancestry individuals with CKD in northern Tanzania, we successfully genotyped three risk variants of the *APOL1* G1 or *APOL1* G2 loci. We did not observe a significant difference in the frequency of *APOL1* G1 and *APOL1* G2 risk alleles compared with previously-reported estimates from ethnically Chagga populations with unknown CKD status from the Kilimanjaro Region in northern Tanzania. We demonstrated a willingness to participate in genetic testing in this community-based population with CKD, with most participants agreeing to genetic testing, and we also demonstrated the feasibility of genotyping *APOL1* risk alleles from dried-blood spot samples on filter cards obtained in the field.

The frequency of *APOL1* G1 and G2 may be as high as 20–40% in several populations from Ghana and Nigeria, with a west-east continental cline for both *APOL1* G1 and G2 risk alleles [6, 12, 14, 29]. These two countries also appear to have among the largest burdens of CKD in sub-Saharan Africa [3]; however, the relative importance of *APOL1* risk alleles in contributing to the development or progression of CKD in African populations is not well known [30]. In central Africa, where the frequency of *APOL1* risk alleles varies, with some populations having estimates as high as 10–16%, the prevalence of CKD ranges from 6–20% [3, 12, 29]. Despite ongoing efforts (*e*.*g*. the H3AFRICA Study), in east Africa where the high burden of CKD is becoming more apparent, the relationship of *APOL1* to CKD is not widely understood [3, 6, 31–33].

**Fig 1:**
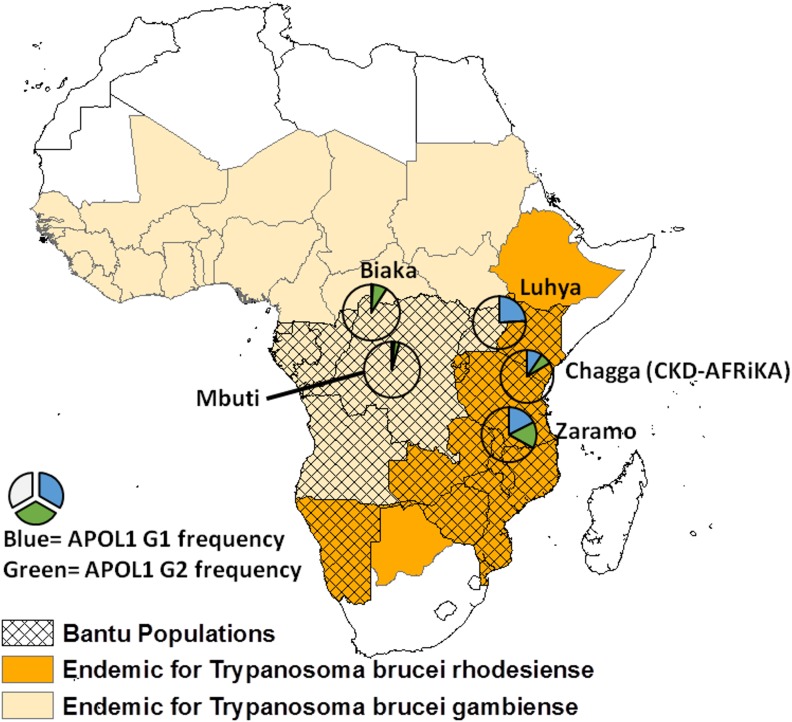
Reported frequencies of *APOL1* G1 and G2 risk alleles for several Bantu populations across sub-Saharan Africa.

To our knowledge, our study is among the first to explore *APOL1* risk allele status among an east African community-based sample of individuals with known CKD. Despite shared Bantu ancestry, compared with other central African Bantu populations we observed a significantly higher frequency of the *APOL1* risk alleles in our east African study population with CKD (**Fig 1**) [23–28]. At the same time, we also observed a significantly lower frequency in our study population compared with other east African Bantu populations, where *Trypanasoma brucei rhodesiense* is most endemic [13]. These observations demonstrate the complex relationship between genetic variation across *APOL1*, ethnic relationships, and *Trypanasoma* exposure in the context of CKD, and they highlight the need for more studies taking these factors into account [17].

We noted several limitations of our pilot study. With respect to one SNP (rs60910145) we observed slight deviation from Hardy-Weinberg equilibrium, which may be from several reasons including selection due to differential resistance to Trypanosomal exposure, non-random mating, or genetic drift in the population at-large, but because of our small sample size we are limited in our ability to more definitively characterize the deviation. Also, in our study sample we observed a higher prevalence of early-stage, proteinuric CKD. In other populations, *APOL1* risk alleles can cause more progressive or more severe CKD phenotypes; therefore, it is not clear to what extent survivor bias or ascertainment bias could affect our findings. Finally, the willingness to participate in genetic testing from individuals with more advanced CKD in this population still needs further exploration. Selection bias resulting from non-response must also be considered, and to reduce non-response rates, we attempted a minimum of two additional visits during off-hours (evenings and weekends) and located participants through phone calls and home-visits when necessary [17]. In this pilot study, we did not genotype individuals without CKD, and although the reference population we used for comparison was similar with respect to ethnicity and location, we were unable to make comparisons for other potentially important confounding variables and many differences may still exist. Similarly, our pilot study was meant to be exploratory in nature, and caution should also be applied when making comparisons with other populations.

In conclusion, in individuals with CKD from northern Tanzania, we demonstrated the feasibility of genotyping *APOL1* risk alleles in a community-based cohort. We successfully genotyped three risk variants from DNA extracted from filter card papers that had been obtained in the field, and we demonstrated a willingness to participate in *APOL1* genotyping studies. These findings suggest future genetic studies of kidney disease would be well-received, and they may inform more extensive field studies into the genetics of CKD in similar populations across the region.

## Conflicts of interest

The authors declare that they have no competing interests. The results in this paper have not been published previously in whole or part, except in abstract format.

## Supporting information

S1 Fig Allelic discrimination cluster plots for *APOL1 G1* and G2 risk variants, (**a**) rs71785313; (**b**) rs60910145; and (**c**) rs73885319.
(TIF)
