# Supplementary figures and images for: APOL1 risk alleles among individuals with CKD in Northern Tanzania: A pilot study
Source: PLoS One. 2017 Jul 21;12(7):e0181811. doi: 10.1371/journal.pone.0181811 (PMC5521837; doi:10.1371/journal.pone.0181811)

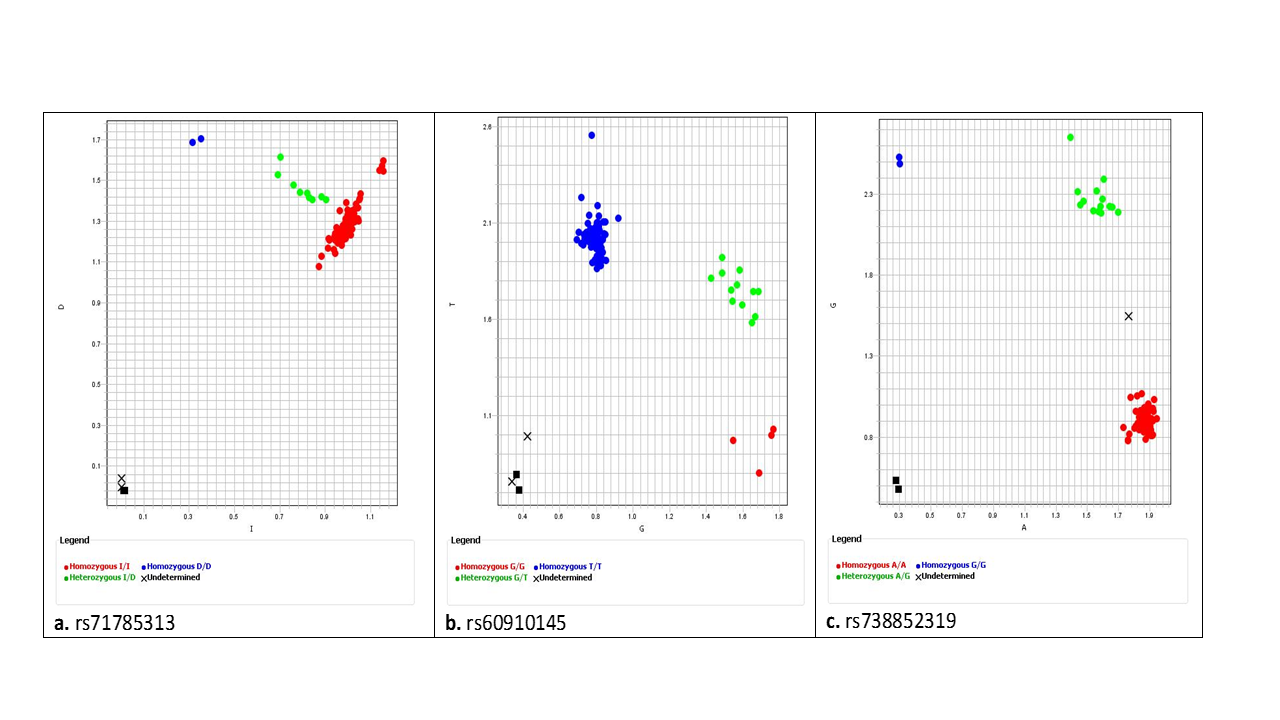

Supplement: S1 Fig — Allelic discrimination cluster plots for APOL1 G1 and G2 risk variants, (a) rs71785313; (b) rs60910145; and (c) rs73885319. (TIF) [file pone.0181811.s001.tif]
